# Non-apoptotic Fas (CD95) Signaling on T Cells Regulates the Resolution of Th2-Mediated Inflammation

**DOI:** 10.3389/fimmu.2018.02521

**Published:** 2018-11-01

**Authors:** Jesse W. Williams, Caroline M. Ferreira, Kelly M. Blaine, Crystal Rayon, Francisco Velázquez, Jiankun Tong, Marcus E. Peter, Anne I. Sperling

**Affiliations:** ^1^Committee on Molecular Pathology and Molecular Medicine, Chicago, IL, United States; ^2^Department of Medicine, Section of Pulmonary and Critical Care Medicine, Chicago, IL, United States; ^3^Department of Pathology, University of Chicago, Chicago, IL, United States; ^4^Feinberg School of Medicine, Northwestern University, Chicago, IL, United States

**Keywords:** allergy, Asthma, Eosinophilia, Apoptosis, Fas-FasL, Th2 cells

## Abstract

Fas (CD95/APO-1) and its ligand (FasL/CD95L) promote the resolution of type 2 lung inflammation and eosinophilia. We previously found that Fas-deficiency on T cells, but not eosinophils, delayed resolution of inflammation. However, Fas can signal both cell death and have a positive signaling function that can actually activate cells. In this study, we investigated whether Fas-induced death or Fas-activated signaling pathways promote resolution of allergic lung inflammation. By increasing T cell survival through two Fas-independent pathways, using Bim-deficient T cells or Bcl-x_L_ overexpressing T cells, no differences in resolution of Th2-mediated inflammation was observed. Furthermore, Th2 cells were inherently resistant to Fas-mediated apoptosis and preferentially signaled through non-apoptotic pathways following FasL treatment. Utilizing Fas-mutant mice deficient in apoptotic but sufficient for non-apoptotic Fas signaling pathways, we demonstrate that non-apoptotic Fas signaling in T cells drives resolution of Th2-mediated airway inflammation. Our findings reveal a previously unknown role for non-apoptotic Fas signaling on Th2 cells in the induction of resolution of type 2 inflammation.

## Introduction

Atopic asthma is supported by a type 2 immune response mediated by expansion of T helper type 2 (Th2) cells reactive against ordinarily innocuous allergens (1, 2). Th2 cells are known to promote pathologic responses associated with asthma, including eosinophilic infiltrate, mucus production, airway constriction, and antibody responses. While increased Th2 cell recruitment to the lungs drives features of allergic responses, it is possible that defective resolution of type 2 responses in asthmatics could also account for prolonged airway disease and contribute to disease phenotypes. The observation that those who suffer from intermittent or persistent asthma have elevated numbers of antigen-experienced T cells and eosinophils in sputum samples, even when samples are collected between exacerbations, supports the premise that asthma may be, in part, driven by defects in resolution of Th2 responses (3). Expanding on the known mechanisms involved in resolving established inflammation in the airways may complement current therapeutic approaches that aim to inhibit the initiation of allergic inflammation, and instead focus on developing methods to end chronic inflammation.

Fas, a member of the tumor necrosis factor (TNF) receptor family, has been broadly studied for its function during development and ability to regulate both ligand-mediated and activation-induced cell death (AICD) in a variety of cells. Asthmatic patients may have defects in Fas-mediated signaling, which could contribute to delayed resolution of inflammation. Following exposure to allergen, FasL expression was augmented in asthmatic patients (4). Low mRNA and surface expression of Fas on pulmonary T cells was associated with persistent inflammatory cell infiltrates in the airways of asthmatics, and their pulmonary lymphocytes were shown to be less sensitive to Fas-mediated apoptosis (5, 6). Similarly, asthmatic patients demonstrated an increased number of cells expressing the anti-apoptotic molecule Bcl-2 compared to normal control subjects, and the expression of Bcl-2 correlated with severity of asthma (7, 8). Together, these data suggest Fas may be important for the regulation of Th2-mediated inflammation.

Fas can initiate two primary signaling pathways: one inducing apoptosis and one promoting a non-apoptotic signaling cascade (9–11). In the initiation of apoptosis, Fas ligation causes a conformational change which allows binding of signaling molecules (including FADD, cFLIP, procaspase-8) to the intracellular C-terminal signaling death domain (12). Recruitment of these proteins along with caspase-8 forms the death-inducing signaling complex (DISC) that induces receptor internalization. Once receptors are internalized, apoptosis can be activated by direct caspase activation or indirectly through a mitochondria-mediated activation pathway. Fas-mediated non-apoptotic signaling is involved in a variety of signaling pathways independent of the death-promoting pathway (13). Fas signaling through FADD-adaptor protein triggers a MAPK signaling cascade to induce NFκB translocation, thereby augmenting cell proliferation and mobility (14–17). Further, non-apoptotic Fas signaling promotes tumor cell growth and invasion (18, 19). The mechanisms that regulate whether Fas-mediated death or non-apoptotic signaling are induced remain to be fully understood.

We previously found that Fas and FasL expression on T cells are necessary for normal kinetics of resolution of Th2-mediated airway inflammation (20, 21). Herein, we investigate the signaling pathway utilized by Fas on T cells during such inflammation. We first tested whether Fas functioned by increasing T cell survival, thereby extending inflammation. However, increasing T cell survival through Fas-independent mechanisms was not sufficient to promote a delay in resolution. Further, Th2 cells are innately resistant to Fas-mediated apoptosis (22) and preferentially signal through non-apoptotic pathways, suggesting that Fas-mediated apoptosis was not a key factor in the extended inflammatory response. Using a mouse model in which apoptotic signaling through Fas on T cells was blocked, we found that the intact non-apoptotic signaling through Fas was sufficient to promote the normal resolution of Th2-mediate airway inflammation. These data expand our understanding of Fas on T cells and identify a new paradigm for the capabilities of non-apoptotic vs. apoptotic Fas signaling during immune responses.

## Results

### Increasing T cell survival is not sufficient to prolong eosinophilic inflammation

**Figure 1:**
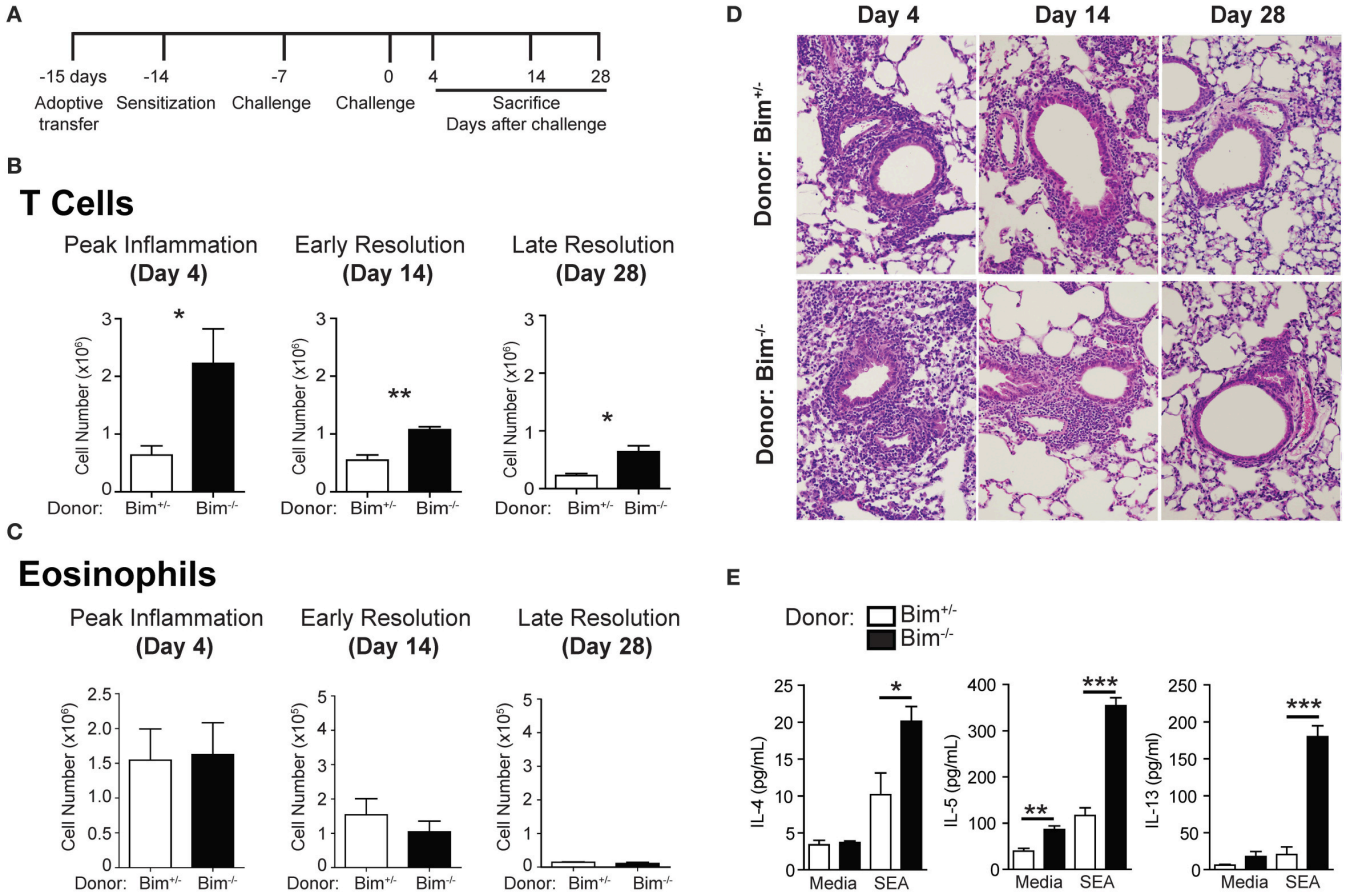
Increasing T cell survival through Bim-deficiency is not sufficient to induced delayed resolution of inflammation. **(A)** Protocol for adoptive T cell transfer, sensitization, and challenge. *Bim*^−/−^ or WT T cells were transferred into *Rag*^−/−^ mice which were then sensitized and challenged. Mice were sacrificed on day 4, day 14, or day 28 after challenge and assayed for **(B)** T cell and **(C)** eosinophil infiltration into the lungs. **(D)** H&E staining of histologic sections from the lungs of sensitized and challenged mice. **(E)** T cells from the lungs at day 14 after challenge were restimulated with media or SEA and assayed for Th2-cytokine secretion. Data are representative of three independent experiments for each time point. For each independent experiment, *n* ≥ 5 mice per group were used (^*^p ≤ 0.05, ^**^p ≤ 0.01, ^***^p ≤ 0.001).

Our previous studies found that Fas-positive T cells promote the resolution of Th2-induced inflammation (20, 21). To test the possibility that these findings were due to changes in T cell survival, we examined whether increasing T cell survival through Fas-independent mechanisms could lead to delays in the resolution of inflammation (23). Bim is a regulatory protein important for the initiation of apoptosis in lymphocytes (24, 25). Adoptive transfer of *Bim*^−/−^ T cells into *Rag*^−/−^ mice (Bim^−/−^>Rag^−/−^) followed by sensitization and challenge resulted in a dramatic increase in the number of T cells having infiltrated into the lungs of mice at all-time points examined (Figures 1A,B). However, this did not correlate with changes in the numbers of eosinophils which had infiltrated into the lungs of these mice (Figure 1C). Similarly, histologic sections from these mice did not show any significant changes in gross pathology when examined at resolution stage of our model (Figure 1D). Th2 cytokine production by re-stimulated T cells from Bim^−/−^>Rag^−/−^ lungs was increased, but augmenting these cytokines alone was not sufficient to drive persistent eosinophilia (Figure 1E).

**Figure 2:**
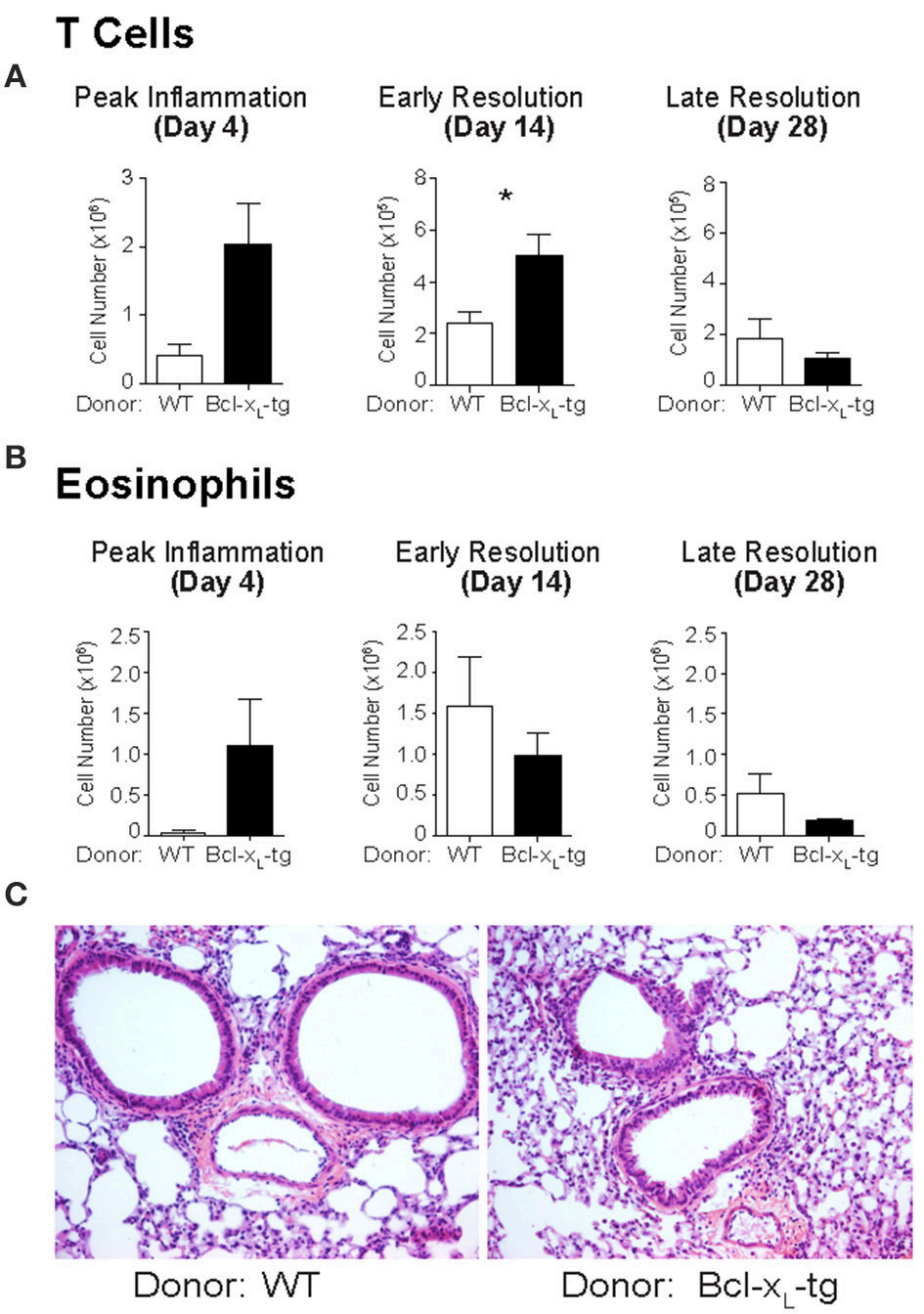
Bcl-x_L_ transgenic T cells promote T cell survival but do not affect eosinophilia in the lungs following sensitization and challenge. Bcl-x_L_ transgenic T cells were transferred into *Rag*^−/−^ mice then sensitized and challenged. Mice were sacrificed on day 4, day 14, or day 28 after challenge and assayed for **(A)** T cell and **(B)** eosinophil infiltration into the lungs. **(C)** H&E staining of histologic lung sections of sensitized and challenged mice at day 14. Data are representative of three independent experiments for each time point with *n* ≥ 5 mice used per group for each experiment (^*^p ≤ 0.05).

We next sought to increase T cell survival through overexpression of the anti-apoptotic protein Bcl-x_L_. Similar to the *Bim*^−/−^ T cells, Bclx_L_ Tg T cell transfer into Rag^−/−^ mice increased the numbers of T cells in the lungs at days 4 and 14 after sensitization and challenge (Figure 2A). Bclx_L_ Tg T cells also failed to influence the numbers of eosinophils recruited to the lungs, nor did they change the gross histologic analysis compared to controls (Figures 2B,C). Together, these data suggest that increasing anti- or pro-apoptotic pathways in T cells, independent of Fas expression, do not affect the resolution of Th2 mediated responses.

### Fas preferentially signals through non-apoptotic pathways on Th2 cells

**Figure 3:**
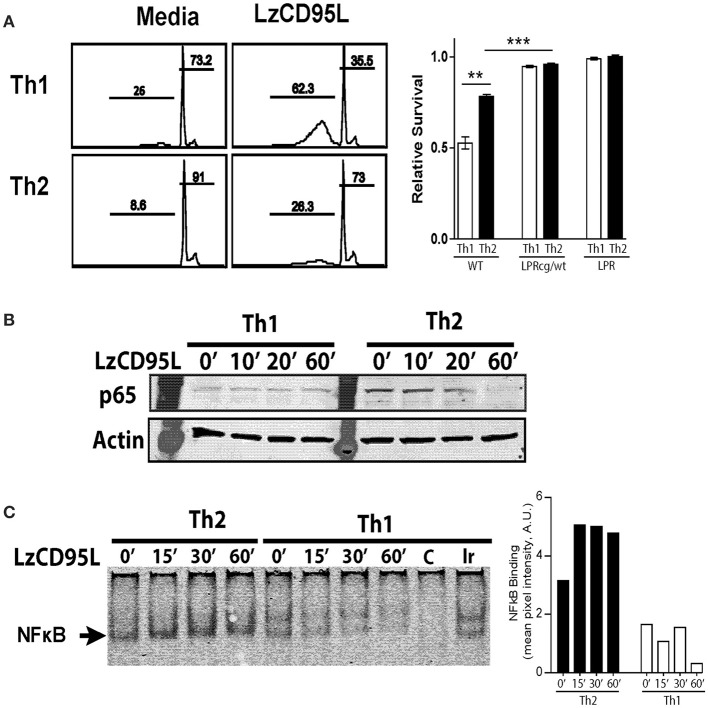
Th2 cells preferentially signal through non-apoptotic Fas pathways following FasL treatment. *In vitro* skewed Th1 and Th2 cells from WT, LPRcg/wt, and LPR mice were treated with LzCD95L and assayed for induction of apoptosis 4 h later by propidium iodide staining. **(A)** Representative PI staining from WT Th1 and Th2 cells showing apoptotic cells in sub-G1, and relative survival rate of T cells from different mice following LzCD95L treatment. **(B)** WT Th1 and Th2 cells were treated with LzCD95L and assayed for cytoplasmic levels of p65 by western blot. **(C)** WT Th1 and Th2 cells were treated with LzCD95L and assayed for nuclear NFκB binding activity by EMSA. Densitometry measurements (right) are displayed as mean pixel intensities in arbitrary units. Data are representative data from three or more independent experiments. In panel **(A)** apoptosis assays included 5 replicated for each independent experiment (^**^p ≤ 0.01, ^***^p ≤ 0.001).

Effector T cell subsets Th1, Th17, and Treg cells are susceptible to Fas-mediated death (26–28). However, the susceptibility of Th2 cells to Fas-mediated apoptosis remains controversial. Even when similar methodologies were used, multiple studies have conflicting conclusions in terms of how Th2 cells respond to Fas-induced apoptosis (27, 29, 30). Unlike previous studies that used antibody for Fas ligation, here we utilized a leucine zipper FasL (LzCD95L) (31) for ligation of Fas on T cells. LzCD95L mimics the membrane bound form of FasL and has been shown to be an efficient inducer of apoptosis and Fas signaling (9). We skewed Th1 and Th2 cells *in vitro*, and treated with LzCD95L to assay for the initiation of apoptosis. As expected, Th1 cells showed a dramatic sensitivity with almost 50% of total cells having DNA fragmentation as measured by a sub-G1 DNA peak (Figure 3A). Skewed Th2 cells were much more resistant to Fas-mediated apoptosis compared to Th1 cells, developing reduced nuclear content loss following treatment. LzCD95L induced death signaling directly through Fas, since neither Fas-deficient (LPR) nor Fas-mutant (LPRcg/wt) cells, showed sensitivity to LzCD95L treatment. These observations support the hypothesis that Th2 cells are resistance to Fas-mediated apoptosis.

We measured NFκB p65 translocation after Fas-signaling to determine whether Th2 cells can signal through Fas non-apoptotic mechanisms. *In vitro* stimulation with LzCD95L resulted in the loss of cytoplasmic NFκB p65 in Th2 cells, suggesting translocation into the nucleus following treatment (Figure 3B). Th1 cells expressed very little p65 and protein levels in the cytoplasm did not change following treatment. Further, LzCD95L induction of nuclear translocation of NFκB was tested by electromobility shift assay (EMSA). As has been previously reported, Th2 cells had augmented nuclear NFκB compared to Th1 cells at baseline (26). Following LzCD95L treatment, Th2 cells developed an increased amount of nuclear NFκB when compared to untreated control Th2 cells (Figure 3C). Together these findings suggest that Th2 cells are resistant to Fas-mediated apoptosis and preferentially signal through non-apoptotic mechanisms in response to FasL engagement.

### Non-apoptotic fas signaling on t cells drives resolution of th2-mediated airway inflammation

**Figure 4:**
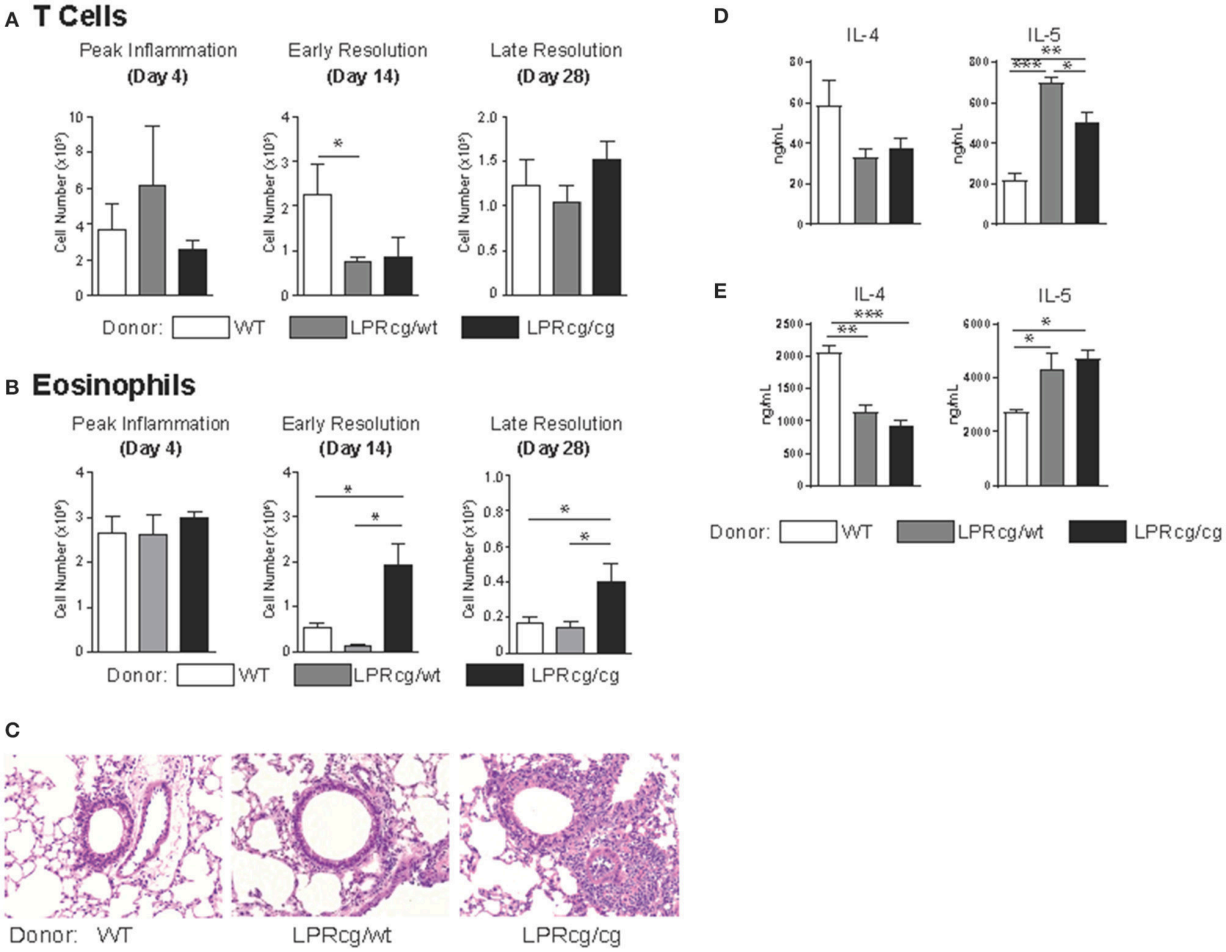
Non-apoptotic Fas signaling in T cells is sufficient for the normal resolution of Th2-mediated inflammation. T cells from the indicated strains were adoptively transferred into *Rag*^−/−^ mice which were then sensitized and challenged then assayed for numbers of **(A)** CD4 T cells and **(B)** eosinophils in the lungs at days 4, 14, and 28 after the final challenge. **(C)** H&E stains of lung sections of sensitized and challenged mice at day 14. Total lung cells were co-cultured with irradiated splenocytes with SEA antigen **(D)** or anti-CD3 antibody **(E)** for T cell cytokine production. **(A–C)** Data are representative of three independent experiments for each time point with *n* ≥ 5 mice per group for each experiment. **(D,E)** data are representative of two independent experiments with *n*- of 3 or 4 mice per group (^*^p ≤ 0.05, ^**^p ≤ 0.01, ^***^p ≤ 0.001).

To address whether non-apoptotic Fas signaling in Th2 cells plays an important role in airway inflammation resolution, we utilized Fas-mutant mice with a point mutation in the death domain of Fas, LPRcg mice (32). When homozygous for the mutation, LPRcg/cg mice are deficient for apoptotic and non-apoptotic signaling similar to the Fas-deficient LPR mice. Interestingly, it has been shown that heterozygous LPRcg/wt mice maintain defects in apoptotic signaling, but are sufficient for induction of non-apoptotic Fas-signaling (33). Utilizing these mice, we asked whether non-apoptotic Fas signaling in T cells is sufficient for promoting resolution of Th2-mediated airway inflammation. T cells from littermate WT, LPRcg/wt, and LPRcg/cg mice were adoptively transferred into *Rag*^−/−^ mice and then sensitized and challenged. Interestingly, CD4 T cells were either equivalent or reduced in the apoptotic-deficient mice, which is strikingly different from what was observed in the *Bim*^−/−^ or Bclx_L_-Tg experiments (Figure 4A). However, eosinophilia was augmented in LPRcg/cg>*Rag*^−/−^ mice at both day 14 and day 28 (Figure 4B). In contrast, LPRcg/wt>*Rag*^−/−^ mice showed equal or even enhanced eosinophil clearance at day 14 and day 28 compared to littermate WT>*Rag*^−/−^ controls (Figure 4B). Histologic examination supported these findings (Figure 4C). When T cells were restimulated with SEA antigen or anti-CD3 antibody in the presence of irradiated APCs, T cells from Fas-mutant mice secreted similar levels of IL-4, IFNγ, and IL-17, but had elevated IL-5 production (Figures 4D,E, and data not shown). However, lung T cells did not produce sufficient cytokines for detection when isolated directly from lungs and plated in media for ELISA, suggesting that it is possible that Fas-mutant Th2 cells are not actively producing more cytokines in the lungs at day 14 following challenge. This result is also in accordance with data from Figure 1, supporting the hypothesis that at later stages of inflammation, Th2 cytokine levels are not regulating the chronicity of disease. Overall, these results demonstrate that non-apoptotic Fas-signaling in T cells is sufficient to promote the normal resolution of allergic airway inflammation in mice.

While Th2 cells are the primary T cell type present in the airways of sensitized and challenged mice, Treg cells have also been implicated in the regulation of inflammation in the lungs (34, 35). To test whether Tregs from Fas-mutant mice may have intrinsic defects in their ability to suppress inflammatory responses, we performed an *in vitro* suppression assay on FACS sorted Treg cells co-cultured with wild type T effector cells. We observed efficient suppression from Tregs regardless of whether they were from WT or Fas-mutant strains (Supplemental Figure 1). These data suggest that Treg effector function is not directly regulated through Fas-signaling pathways.

## Methods

### Mice

*Rag*^−/−^ (B6.129S7-*Rag1*^*tm1Mom*^/J), *Bim*^−/−^ (B6.129-Bcl21ll tm1.1Ast /J), and LPR (B6.MRL-*Fas*^*lpr*^/J) were purchased from The Jackson Laboratory (Bar Harbor, Maine USA). Bcl-x_L_ Tg; Lck-p-Bcl-xL Tg (B6.Cg-Tg(LCKprBCL2L1)12Sjk/J) mice (36) were a gift from Dr. Jeff Rathmell (Vanderbilt University). LPRcg (MRL/MpJ-Faslprcg/Faslprcg) mice were a gift from Dr. Marcus Peter (Northwestern University), and backcrossed to C57BL/6 for 10 generations. FoxP3^gfp^ reporter mice (B6.Cg-Foxp3tm2Tch/J) were obtained from the Jackson Laboratories, and crossed to LPRcg strain. All mice were bred and housed in specific pathogen-free facilities maintained by the University of Chicago Animal Resource Center. Studies described conform to the principles set forth by the Animal Welfare Act and the National Institutes of Health guidelines for the care and use of laboratory animals in biomedical research.

### Flow cytometric analysis

In brief, 5 × 10^5^ cells were suspended in 50 μl of FACS buffer (1x PBS containing 0.1% sodium azide and 1% BSA), blocked with anti-CD16/32 (2.4G2), and labeled with specific antibodies. Antibodies used include anti-CD3 (145-2C11), -CD4 (GK1.5), -CD8 (53-6.7), -Ly6G (Gr1) (BD Biosciences), and -CCR3 (83101) (R&D Systems). Flow cytometric analysis was performed on an LSR Fortessa (BD Biosciences), and data were analyzed with FlowJo software (Tree Star Inc.).

### *S. mansoni* allergic inflammation model

Inactivated *S. mansoni* eggs (5,000), a generous gift from Dr. Joel Weinstock, Tufts University, were administered to mice i.p. at day 0 and animals were challenged intratracheally on day 7 and day 14 with 5 μg of soluble eggs antigen (SEA) in 50 μL PBS (20, 37). Mice were sacrificed on days 4, day 14, and day 28 after the final i.t. challenge. At sacrifice, animals were perfused with 5 mL 1x PBS, then BAL and lungs were collected for analysis. As previously published (38), BAL was performed by lavaging 0.8 ml ice old 1x PBS into the airway via a tracheal cannula and gently aspirating the fluid. The lavage was repeated four times, with < 90% of fluid recovered. For histological analysis, lungs were fixed with formalin. Samples were paraffin embedded and cut sagitally into 5 μM sections. Sections were stained with Hematocylin and Eosin (H&E) or with Periodic Acid Schiff (PAS) by the University of Chicago Histology Core Facility.

### Cytokine secretion

Lungs from mice were isolated, cut with scissors into small pieces, and digested with 2 mg/mL Collagenase IV at 37-degrees Celsius for 45 min. Tissue was strained through nylon filters to isolate single cell suspension. T cells from lungs of mice were restimulated by co-culture irradiated (24 gray) B6 splenocytes that were loaded with antigen (1 μg/mL SEA) and were incubated at 37°C in complete medium DMEM, 10% fetal bovine serum (HyClone Characterized, Thermo Fisher, Cat# SH30396, Lot# KTH31760), 1% β-mercaptoethanol, and antibiotics. After 48 hrs, plates were directly frozen without separating supernatant from stimulated cells. Total cytokine production was measured using a Millipore Multiplex bead array following manufacturer's instructions and analyzed by a Luminex (Bio-Rad) reader.

### Treg suppression assay

Tregs from FoxP3^gfp^ reporter mice crossed to LPRcg mutant mice were FACS sorted, then co-cultured with CFSE-labeled FACS sorted wild type FoxP3^gfp^-negative T effector cells. Cells were plated with irradiated splenocytes and anti-CD3 (2C11) antibody to induce effector cell proliferation. CFSE dilution was measured by flow cytometry at day 4 of culture. Forty thousand of effector cells were cocultured with 0, 1,000, 2,000, 4,000, and 16,000 Treg cells to measure suppressive ability of Treg cells.

### Apoptosis assays

Apoptosis of cells was measured using the Nicoletti propidium iodide staining protocol (39). These experiments were performed 4 h post treatment with 200 ng/mL LzCD95L (Marcus Peter Lab, Northwestern University).

### Electromobility shift assay (EMSA)

Nuclear extract from LzCD95L treated cells was incubated with IR-labeled NFκB oligonucleotide probe (Li-cor #829-0724) following manufacturer's instructions. Samples were run on a 4% native polyacrylamide gel at 10 V/cm for 60 min and directly imaged (Li-cor Odyssey). Mean pixel intensity of bands was performed on Image J software.

### Western blotting

Cells were lysed [25 mM HEPES (pH 7.5)], 150 mM NaCl, 1% Triton X-100, 2 mM EDTA, 2 mM EGTA, 10% glycerol, 1 mM NaF, 200 μM Na-orthovanadate, and protease inhibitor cocktail (Sigma Aldrich catalog #S8830, 1 tablet for 100 mL lysis buffer) and insoluble material was removed by centrifugation at 20,000 × g for 5 min. Cleared lysates were boiled in Laemmli buffer for 5 min. Samples were subjected to polyacrylamide gel electrophoresis, and analyzed by blotting with primary antibodies against p-65 (Santa Cruz), and β-actin (Sigma-Aldrich), followed by corresponding secondary antibodies conjugated with IR-dye and imaged using Li-cor Odyssey and software.

### T cell enrichment

Nylon wool columns were incubated with PBS + 5% FBS for 1 h at 37°C to decrease background binding. Cells were incubated at 37°C on the column for 45 min and then 30 mL of 5% complete media was passed through the column to enrich T cells. Non-adherent cells were collected and assayed for T cell purity of < 90%.

### Statistical analysis

Statistics were calculated using the GraphPad (Prism 4) software with unpaired students two-tailed *T*-tests, (^*^p < 0.05, ^**^p < 0.005, ^***^p < 0.0005, ns = not significant). Error bars on all graphs represent mean ± standard error (SEM).

## Discussion

In this study we addressed the mechanisms by which Fas regulates the resolution of experimental asthma in mice. We identified an *in vivo* function for non-apoptotic Fas signaling in normal T cells, independent of pathways regulating cell survival. The importance of non-apoptotic Fas-signaling in other cell types such as tumor cells has been observed, but our study represents the first to investigate the role of non-apoptotic Fas signaling in maintaining immune homeostasis.

Non-apoptotic pathways have been implicated in a wide variety of cell types outside of just T lymphocytes; including fibroblasts (40) astrocytes (41), neurites (14), and a number of cancer models (11, 42–45). While others have argued that strength of signal as a key regulator of apoptotic or non-apoptotic Fas signaling, our data support the notion that some cells (particularly Th2 cells) are intrinsically programed for non-apoptotic signaling (19, 33). Further, whether these intrinsic programs are modulated by different antigen exposures (i.e., viral vs. bacterial infections) or epigenetically by chromatin remodeling have yet to be thoroughly vetted. As a recent example, T cells from patients with Human T-cell lymphotropic Virus 1 (HTLV-1) infection adopted preferential non-apoptotic Fas signaling. This non-apoptotic signaling through Fas resulted in enhanced T cell proliferation, activation, and a more rapid onset of infection (46).

In tumor cell lines, Fas mediated non-apoptotic signaling pathways through MAP kinase and NF-κB activation, have been shown to drive invasiveness (44). This non-apoptotic signaling through Fas was enhanced in cells that have a single allelic mutation of the Fas gene which completely blocked apoptotic signaling through Fas (33). This observation is analogous to the results obtained with the LPRcg/wt mouse model in our study where we observed that a single allele mutation in Fas was sufficient to inhibit death-inducing pathways, but enabled positive signals to still occur downstream of the receptor. These observations led to the conclusion that two functional Fas alleles are necessary for the induction of apoptosis, and that the presence of a mutant allele could lead to growth and cell survival, which in the case of tumors is an undesirable outcome (33, 44). Consequently, blockade of Fas signaling in a tumor line led to decreased migration and activation of JNK, NFκB, p38 pathways (42). Finally, non-apoptotic Fas signal via both ERK1/2 and p35 promotes neurite outgrowth (14). Thus, adoption of non-apoptotic signaling pathways in cells that are meant to be regulated by Fas-mediated apoptosis may lead to scenarios of disease states.

We observed that Th2 cells had cell-intrinsic resistance to Fas-mediated apoptosis, and in fact had induced NFκB activity following FasL treatment. Resistance of Th2 cells to FasL-mediated killing may be due to Th2 cells having augmented FLIP, TRAIL, and NFκB expression at baseline when compared to Th1 cells (26). Our *in vivo* data further suggested that this preference for non-apoptotic signaling represents a predisposition for Fas signaling on Th2 cells and contributes to functional outcomes in lung inflammation. However, whether cells that utilize non-apoptotic signaling are intrinsically predisposed, or whether the context of ligand exposure (such as microenvironment or ligand conformation) regulates the downstream signaling remains to be thoroughly explored. Previous studies suggested that Fas engagement of membrane-bound FasL vs. cleaved soluble-FasL had different outcomes in Fas-induced cell death and signaling (47, 48). Two animal models were developed; one expressing only soluble FasL and expressing only membrane-bound FasL. Analysis of these mice in a tumor model led to the conclusion that the nature of the FasL isoform could lead to changes in strength of signal and differential signaling outcomes in disease (19). Our data does suggest that even cells with a high degree of similarity, such as CD4 T cells subsets, have dramatically different signaling outcomes when encountering the same form of Fas ligand. In our case, using LzCD95L resulted in observations that each T cell type is skewed for preferential signaling pathways downstream of FasL-engagement. However, it is important to consider that these findings do not exclude the possibility that under some scenarios Th2 cells might undergo a shift in the balance between pro- and non-apoptotic signaling, enabling these cells to now switch to an apoptotic sensitive cell type. Unraveling these possibilities will help elucidate potential targets for intervening in diseases associated with Fas expression and function.

While our data clearly show that T cell-mediated Fas-signaling is necessary for normal resolution of Type-2 inflammation, we were not able to separate the role of Fas-signaling on Th2 cells from other T cell subpopulations. By utilizing an adoptive T cell transfer approach, we were able to create a scenario where T cells were the only cell type possessing the Fas-mutations. However, this would include CD8, as well as other CD4 T effector cells, leaving the possibility that Fas-signaling pathways on non-Th2 cells could be playing a role in Type-2 inflammation resolution. Treg cells are established regulators of inflammation in all parts of the body including lung. We show that Treg suppression was not affected in Fas-mutant mice. In addition, we demonstrate that Fas-signaling on Th2 cells induces non-apoptotic activation instead of cell death. Thus, while this study emphasizes a previously unappreciated role for Th2 cells *in vivo*, limitations exist in our ability to interpret the mechanisms as other T cell subsets may be contributing to effective resolution of inflammation.

In conclusion, using an *in vivo* model of experimental asthma, we show that non-apoptotic Fas signaling on T cells is necessary for resolution of Th2-mediated inflammation. Resolution of inflammation was not dependent on regulatory mechanisms associated with T cell apoptotic pathways or T cell numbers present in the airways. Translating these findings into humans for targeting of Fas pathways in patients may present a variety of challenges and limitations. For instance, as Fas is expressed in most hematopoietic and stromal cells, specific targeting of Fas to the cells of interest may be impossible. Moreover, broadly targeting Fas or FasL pathways by antibodies or recombinant proteins could have unpredictable outcomes: inducing apoptosis in some cells and activation in other cell types, as well as causing organ damage like liver injury (49). However, based on our results, optimization of drug targeting of Th2 cells specifically appear to be a potential mechanism to drive the resolution of Type-2 inflammation. Expanding on our understanding discrete function of Fas on Th2 effector T cells may allow for unique targeting in allergic diseases, specifically asthma and potentially other responses involving Th2 cells.

## Author contributions

AS and MP conceived and supervised the project. JW, CF, and AS wrote the manuscript. JW, CF, KB, CR, FV, and JT performed and analyzed experiments.

### Conflict of interest statement

The authors declare that the research was conducted in the absence of any commercial or financial relationships that could be construed as a potential conflict of interest.
