# Supplementary figures and images for: Non-apoptotic Fas (CD95) Signaling on T Cells Regulates the Resolution of Th2-Mediated Inflammation
Source: Front Immunol. 2018 Nov 1;9:2521. doi: 10.3389/fimmu.2018.02521 (PMC6221963; doi:10.3389/fimmu.2018.02521)

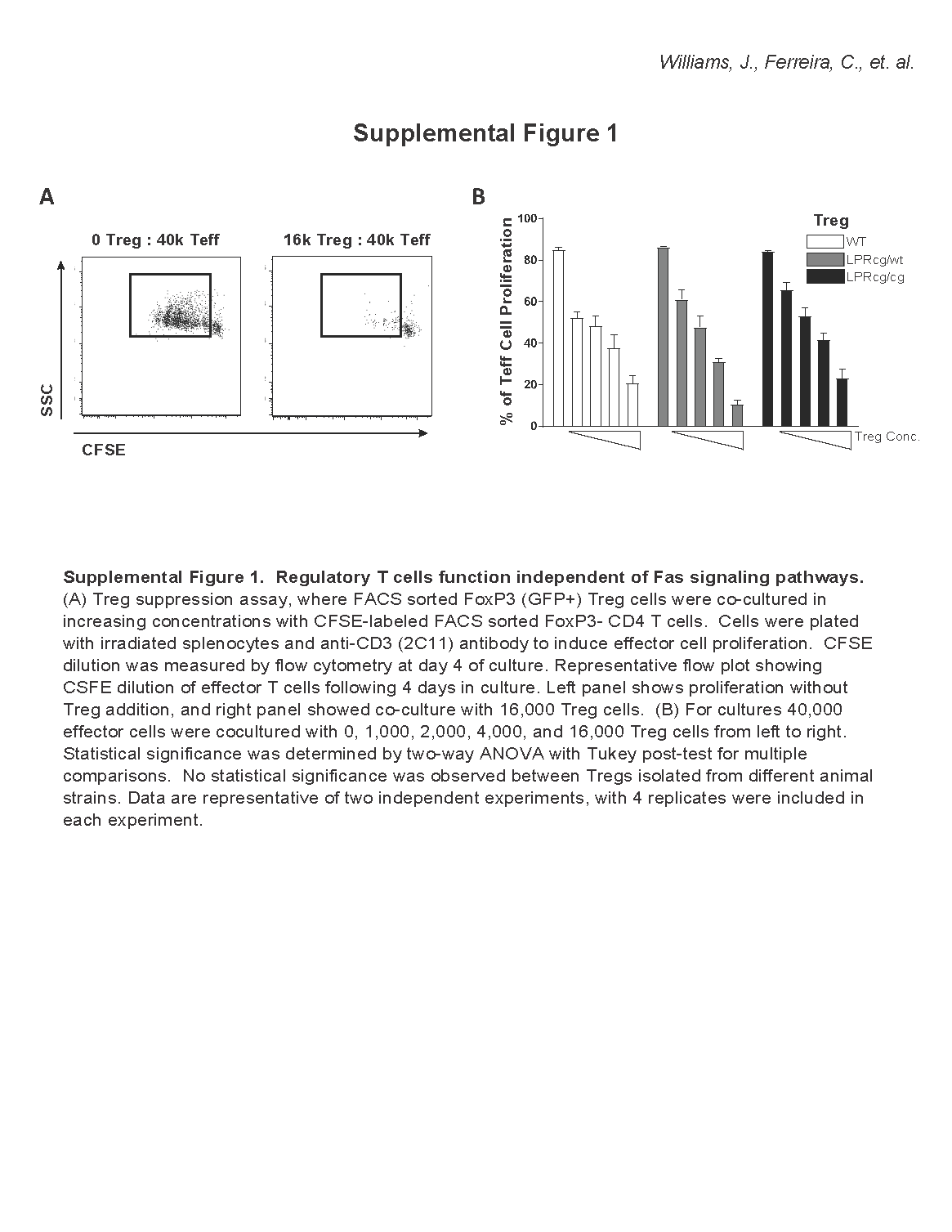

Supplement: Supplementary file 1 [file Image_1.TIFF]
